# Facile Preparation of a Robust and Durable Superhydrophobic Coating Using Biodegradable Lignin-Coated Cellulose Nanocrystal Particles

**DOI:** 10.3390/ma10091080

**Published:** 2017-09-14

**Authors:** Jingda Huang, Siqun Wang, Shaoyi Lyu

**Affiliations:** 1 Research Institute of Wood Industry, Chinese Academy of Forestry, Beijing 100091, China; hjd1015@163.com; 2 Center for Renewable Carbon, University of Tennessee, Knoxville, TN 37996, USA

**Keywords:** superhydrophobic coating, lignin-coated cellulose nanocrystal (L-CNC), mechanical strength, durability

## Abstract

It is a challenge for a superhydrophobic coating to overcome the poor robustness and the rough surface structure that is usually built using inorganic particles that are difficult to degrade. In this study, a robust superhydrophobic coating is facilely prepared by using commercial biodegradable lignin-coated cellulose nanocrystal (L-CNC) particles after hydrophobic modification to build rough surface structures, and by choosing two different adhesives (double-sided tape and quick-setting epoxy) to support adhesion between the L-CNC particles and the substrates. In addition to excellent self-cleaning and water repellence properties, the resulting coatings show outstanding mechanical strength and durability against sandpaper abrasion, finger-wipe, knife-scratch, water jet, UV radiation, high temperature, and acidic and alkali solutions, possessing a wide application prospect.

## 1. Introduction

Wettability is a very important characteristic and has a significant influence on actual applications for a solid material surface. At first found in nature, such as in lotus leaves [1], superhydrophobic surfaces exhibit many outstanding functions, such as self-cleaning [2,3], anti-icing [4], anti-fog [5] and corrosion-resistant [6,7] functions, and have attracted much attention. An artificial superhydrophobic coating can be created on a hydrophilic surface by building suitable roughness and lowering surface free energy. Following decades of development, there have been many reports on the preparation of a superhydrophobic coating applied to different materials surfaces, such as wood [8], clothes [9,10], metal [11,12,13], filter paper [14], glass [15], and plastic [16]. Different preparation methods have also been employed, such as sol-gel [17], immersion [18], electrochemical deposition [19], spraying [20,21], etching [22,23], chemical vapor deposition (CVD) [24], and electrospinning [25]. However, superhydrophobic coatings still suffer from much obsession. In particular, poor abrasion resistance is a recognized challenge and directly limits their further application.

Beyond doubt, robustness has become a hot topic with respect to the study of superhydrophobic coatings. A variety of methods are presented to solve the poor abrasion resistance. For example, in a report by Reference [26], polydimethylsiloxane (PDMS) was used as an interlayer between a superhydrophobic SiO_2_ layer and a glass substrate to supply adhesion. The resulted coating showed good abrasion resistance and could withstand nine cycles of sandpaper (150 grit) abrasion under a weight of 50 g. It was reported [27] that a superhydrophobic coating was prepared by spraying a mixture of SiO_2_ nanoparticles modified with fluoroalkylsilane. The resulting coating was mechanically robust and could withstand 200 cycles of sandpaper (2000 grit) abrasion under a 2-kg force. It could be seen that inorganic particles such as SiO_2_ are generally used as the main materials to build a rough surface structure.

Recently, our research group has made some progress in the preparation of superhydrophobic coatings using biodegradable materials. We successfully prepared a cellulose nanofibril (CNF) superhydrophobic coating by CVD after spraying [28], but the abrasion resistance still requires significant improvement. Additionally, the CVD modification method is not convenient to use in some areas such as building walls or wide panels. Cellulose nanocrystal (CNC) is usually isolated from cellulose fiber via an acid hydrolyzation method [29,30] and stored in the form of an aqueous solution where CNCs could repel each other due to their negative surface charges. Due to their high strength and environmental friendliness, CNCs are often used as a reinforcement material. As is known, the reinforcement effect will be limited, unless individual dried CNC can be evenly dispersed. However, the large quantities of hydroxyls on their surfaces would lead to CNC aggregation after drying, and it will be very difficult to disperse the aggregated CNCs again.

In this research, individual dried CNC is undesired. Instead, the lignin-coated cellulose nanocrystal (L-CNC) particles formed from the L-CNC aggregation are required. Besides being natural biodegradable, L-CNC particles are of high strength due to the high crystallinity of CNCs themselves [31]. A suitable roughness is necessary for a superhydrophobic coating [32]. Unlike some coatings [33,34] where different sizes of particles are needed to build a rough surface structure, the L-CNC particles possess irregular sizes and unsmooth surfaces and could supply suitable roughness by themselves. Also, the L-CNC particle surfaces have many hydroxyl groups and are beneficial to hydrophobic modification. To fix the L-CNC particles to a substrate surface, an adhesive is needed. It was reported that a robust superhydrophobic coating could be prepared using double-sided adhesive or spray adhesive as binders between TiO_2_ particles with hydrophobic modification and substrates [34]. Inspired by this article, in this study, a quick-setting epoxy resin and a double-sided tape are used to supply adhesion.

**Figure 1:**
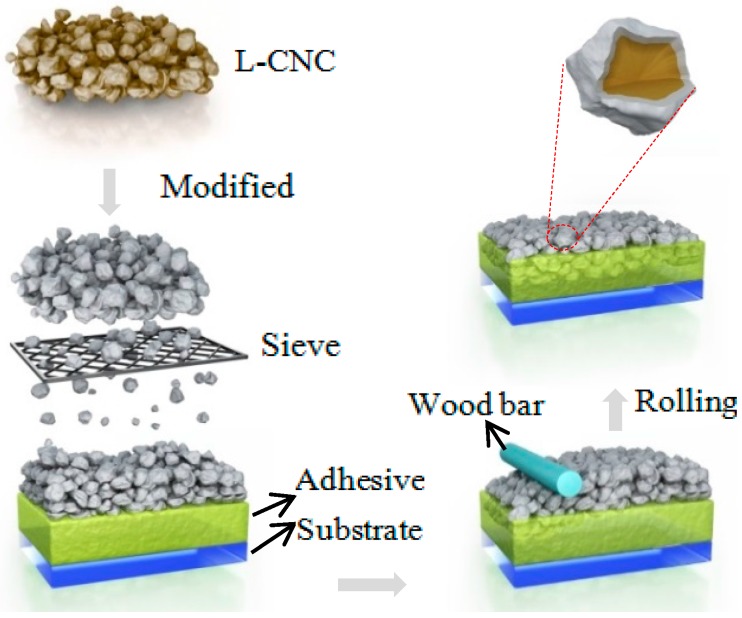
Sketch of the procedure to prepare the composite superhydrophobic coating.

The preparation process is simple, as shown in Figure 1. First, hydrophilic L-CNC particles are modified using a low-energy reagent. And then, the hydrophobic L-CNC particles are completely covered onto the substrate surface, which was previously treated with adhesive. Lastly, a wooden bar is used to press the L-CNC particles onto the adhesive. The resulting coatings show excellent superhydrophobic properties, as well as high mechanical strength and durability. Furthermore, the L-CNC particles are biodegradable, non-toxic, and not harmful to environment, and the quick-setting epoxy or double-sided tape can increase the processing efficiency without requiring a cumbersome process.

## 2. Results and Discussion

### 2.1. Surface Morphology of the Coatings

**Figure 2:**
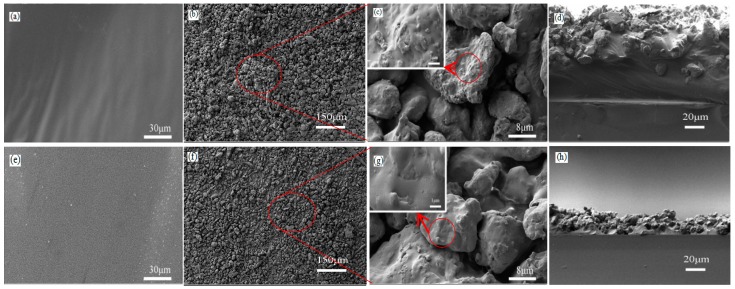
(**a**) Surface morphology of (**a**) the double-sided tape; (**b**) the D coating; (**c**) the L-CNC particles, with a high-magnification image inset; (**d**) the cross-sections of the D coating; (**e**) the surface morphology of the quick-setting epoxy; (**f**) the E coating; (**g**) the L-CNC particles, with a high-magnification image inset; and (**h**) the cross-sections of the E coating.

As shown in Figure 2a, the double-sided adhesive tape surface is smooth and cannot supply enough roughness. When the L-CNC particles are directly scattered onto the double-sided adhesive surface and rolled with the wooden bar, the L-CNC particles/double-sided tape coating (marked as D coating) is formed (as shown in Figure 2b). Under the action of the wooden bar, some of L-CNC particles are entirely buried inside the adhesive and thus lose the function to build surface roughness. Other L-CNC particles are only partially buried in the adhesive. The rest do not make contact with the adhesive. As can be seen from Figure 2c, the L-CNC particles are irregular and have a diameter of approximately 2–20 μm. The high-magnification image shows that the L-CNC particle surface has many nanoscale gaps and protuberances instead of being smooth. Therefore, the L-CNC particle itself is a micro-nano hierarchical structure, which is one of necessary conditions for a superhydrophobic coating. Similar to Figure 2a–c, Figure 2e–g show that the pure epoxy surface is also smooth, and the compounding pattern between the L-CNC particles and epoxy in the L-CNC/epoxy coating (marked as E coating) is the same as that of the D coating. Figure 2d,h show cross-sections of the D and E coatings; the different regions are able to be clearly distinguished between the glass slide, adhesives, and L-CNC particles. The thickness of the D and E coatings is estimated to be approximately 120 μm and 30 μm, respectively.

### 2.2. Wettability

**Figure 3:**
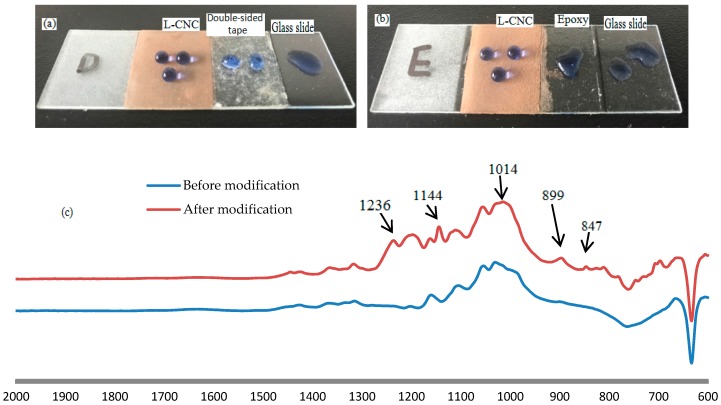
Status of water droplets on (**a**) the untreated glass slide, the double-sided tape, and the glass slide treated with D coating, and (**b**) the untreated glass slide, the epoxy, and the glass slide treated with E coating; (**c**) FTIR spectra of the L-CNC particles before and after modification.

Figure 3a,b show that the untreated glass slide and the adhesive surfaces are hydrophilic and the water droplets (dyed blue with methylene) could spread out, resulting from a large amount of hydroxyl groups in the area. The water droplets on the glass slide treated with modified L-CNC particles could form spheres and rolled off easily with only slight shaking, and the static water contact angle (WCA) of the D coatings reach 163.5° and that of the E coating being 161.2°, while the slide angle (SA) of the D or E coating is less than 10°. This benefits from large amounts of F originating from 1H,1H,2H,2H-perfluorooctyltrichlorosilane (PFTS), which is currently known to have the lowest energy. As is known, it is difficult for liquid to infiltrate a material surface that has a lower surface free energy than the liquid. According to the Cassie wetting state, there is a thin layer of air in the interface between the water and the substrate. Compared with a smooth surface, the actual contact area between water droplets and the solid surface is smaller and there is less adhesion in a superhydrophobic surface, allowing the water droplets to roll off easily.

Figure 3c shows the FTIR spectra of the L-CNC particles before and after modification. The low surface free energy is mainly attributed to the C–F bonds from PFTS. The stretching vibration of the C–F bonds led to two new peaks at 1236 and 1144 cm^−1^. Three new peaks at 1014, 899, and 847 cm^−1^ were caused by the stretching vibrations of the Si–O bonds that were formed from the dehydration of the hydroxyls between the PFTS after hydrolysis and the L-CNC particle surfaces. This suggests that PFTS could be effectively grafted to the L-CNC particle surfaces.

### 2.3. Water Repellence in Air or Oil and Self-Cleaning

**Figure 4:**
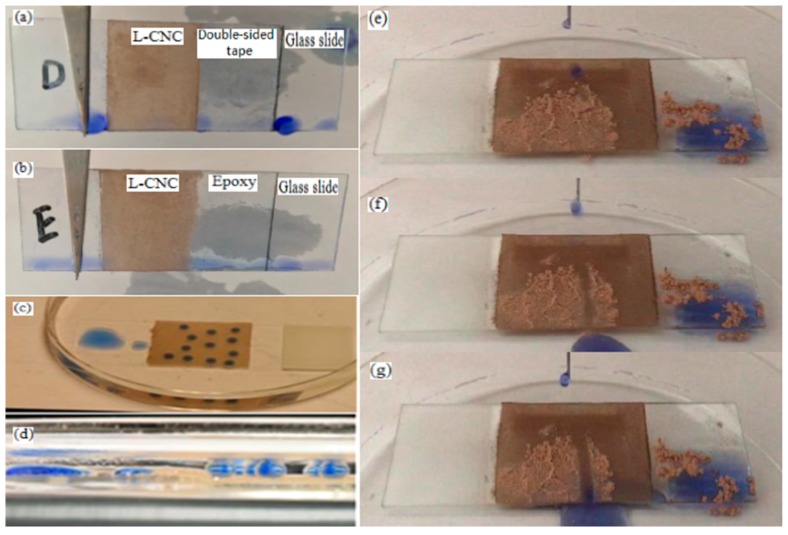
Water repellence properties of (**a**) the D and (**b**) the E coatings; status of the water droplets on the untreated glass slide and the glass slide treated with the D coating from (**c**) a top-down view and (**d**) an eye level view; (**e**–**g**) the self-cleaning test of the D coating.

As shown in Figure 4a,b, when entirely immersed in water and taken out, the untreated parts of the glass slides and the adhesive surfaces clearly present residual water, but there is no contamination in the treated parts with both the D and E coatings. This proves their outstanding water-repellence. This finding is not limited in air, but the coating also shows good water-repellence in oil (n-dodecane), which is of great significance for applications such as bearings and gears [34]. As shown in Figure 4c,d, on the untreated part of the glass slide, water droplets spread out and become a thin layer. However, on the treated part, the water droplets remain spheres without spreading out, even when the coating is polluted by the oil.

Self-cleaning is a very important application for a superhydrophobic surface. As shown in Figure 4e–g, in the untreated part of the glass slide, water droplets spread down instead of rolling away, resulting in larger pollution. However, on the treated part, the water droplets form spheres and roll away. The hydrophilic dust is attached to the rolling water droplets surface and taken away, leaving a clean rolling trace.

### 2.4. Mechanical Strength

#### 2.4.1. Sandpaper Abrasion Test

**Figure 5:**
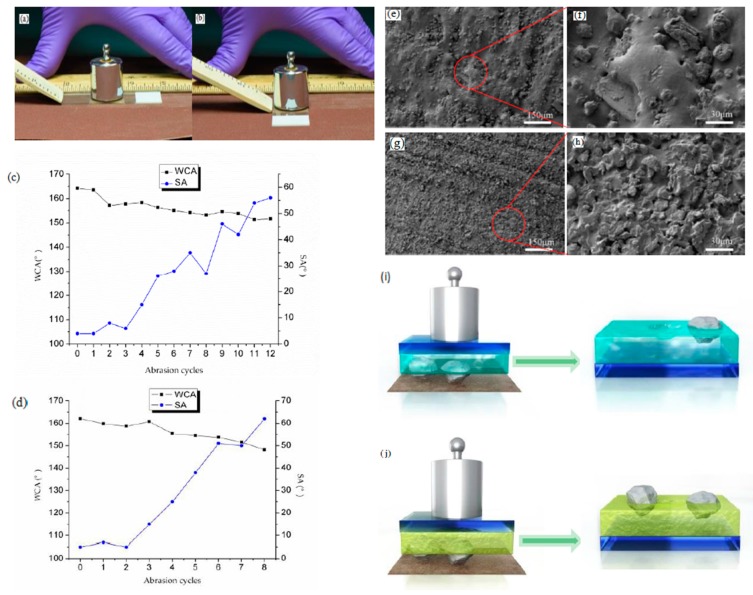
(**a**,**b**) Demonstration of one abrasion cycle; changes curves of WCA and SA of (**c**) the D coating and (**d**) the E coating during abrasion; surface status of (**e**,**f**) the D coating after 12 abrasion cycles and (**g**,**h**) the E coating after eight abrasion cycles; illustration of the L-CNC particles before and after abrasion in the (**i**) E coating and (**j**) the D coating.

Abrasion resistance is a key determinant of working life for a superhydrophobic coating. The sandpaper abrasion test is the most common test method. One abrasion cycle is shown in Figure 5a,b. It is easy to comprehend that if there is no adhesive between the L-CNC particles and the substrates, the L-CNC particles would not be fixed and easily scraped off. In the coating treated with adhesives, the L-CNC particles without contact with adhesive could also be easily removed. So, adhesives are crucial for the stability of the coatings. In addition to adhesives, the thickness of the coatings and the strength of the particles also contribute to the abrasion resistance. In the report by Reference [35], a thin superamphiphobic surface could be created on paper by plasma processing and vapor phase silanization of fluorosilane. In this research, the superhydrophobic coating is thicker, resulting from the large size of the L-CNC particles and the high thickness of the double-sided adhesive and quick-setting epoxy layer. As is known, a surface is defined as superhydrophobic when it exhibits a WCA larger than 150° and an SA smaller than 10° [36]. As shown in Figure 5c,d, after exposure to a 100-g weight and a sheet of sandpaper (320 grit), the D coating could not withstand three abrasion cycles (total 60 cm) and the E coating could not withstand two cycles (total 40 cm) until the SA was larger than 10°. With the increase of the abrasion cycles, the WCA decreases and the SA presents an increasing trend, resulting from the severe damage and wastage of the rough surface structure (as shown in Figure 5e–h).

The abrasion resistance of the D coating is a little better than that of the E coating. This is mainly attributed to the different adhesives used. For the E coating, the adhesive is epoxy resin, which is a type of thermosetting resin with high hardness after fast curing. As shown in Figure 5i, during the sandpaper abrasion, the L-CNC particles in the E coating appear as one of two conditions. One is that the L-CNC particles whose small fraction is buried in the adhesive are easily shaved and uprooted due to insufficient adhesion, leaving a pit on the adhesive surface. The other is that the L-CNC particles whose large fraction is buried in the adhesive are not uprooted. The latter benefits from a higher adhesion and could withstand the impact of the sandpaper, but the particle surfaces would also be abraded. The two conditions would damage the surface structure and cause a change in wettability.

For the D coating, the double-sided tape is a pressure-sensitive adhesive which has high viscosity at room temperature. During the sandpaper abrasion, the D coating also appears in two conditions (as shown in Figure 5j). One is that within a certain number of abrasion cycles, the L-CNC particles whose small fraction is in contact with the adhesive would not be wiped away. Instead, some of them would sink deeper and move forward a certain distance under the impact of the sandpaper due to the high viscosity of the double-sided adhesive. The other is that the L-CNC particles whose large fraction is in contact with the adhesive are not moved a long distance due to a stronger adhesion, but the particle surfaces are also abraded. These two conditions also lead to damage of the surface structure, but the damage is lighter than that of the E coating within the same number of abrasion cycles. This is contributed to the high adhesion and excellent flexibility of the double-sided adhesive. When the D coating is treated with the sandpaper, the double-sided adhesive could transform and reduce some of the sandpaper’s impact. However, for the E coating, the quick-setting epoxy, a thermosetting resin, cannot transform and buffer the sandpaper’s impact. Thus, under the same abrasion conditions, the D coating shows better resistance to abrasion than the E coating.

#### 2.4.2. Finger-Wipe and Knife-Scratch Test

The D coating was used as the tested sample and scraped three times at different areas with a gloved finger. The results show that the superhydrophobic property has no obvious change, which demonstrates the strong micro structure of the coating surface. Upon continuing to scratch the coating with a knife, the coating was not been scraped or torn, which demonstrates that the double-sided adhesive is able to supply high adhesion.

#### 2.4.3. Water Jet Test

**Figure 6:**
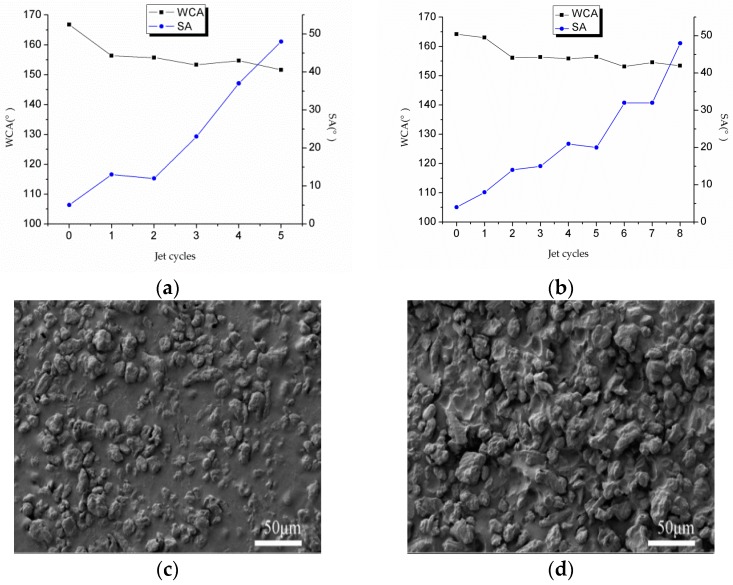
Change curves of the WCA and SA of (**a**) the D coating and (**b**) the E coating in the water jet process; surface morphologies of (**c**) the D coating and (**d**) the E coating after the same five jet cycles.

It is valuable for outdoor applications to imitate heavy rain to scour the D and E coatings. As shown in Figure 6a,b, the WCA shows a decreasing trend and the SA has an increasing trend. After five jet cycles and dried, the D coating exhibits a WCA less than 150°. Figure 6c shows the surface morphology of the D coating after five jet cycles. The L-CNC particles in a unit area are reduced and there are even some large areas of double-sided adhesive exposed. This is because the L-CNC particles with only a small fraction in the adhesive are washed away by the large impact force of the water jet, and the distance between the adjacent L-CNC particles in these areas become wider and thus the surface structure is damaged. It is also possible for water droplets to contact to the hydrophilic adhesive surface, resulting in a decrease of the WCA. However, after the same five jet cycles and drying, the E coating still maintains a WCA larger than 150°. As shown in Figure 6d, there are more L-CNC particles than the D coating in the same unit and until eight jet cycles, after which the E coating does not show a WCA lower than 150°, proving that the quick-setting epoxy is more water-tolerant and could supply stronger adhesion than the double-sided adhesive, which would shrink and make poorer contact with the L-CNC particles due to the water jet.

### 2.5. Durability

#### 2.5.1. UV Radiation Test

**Figure 7:**
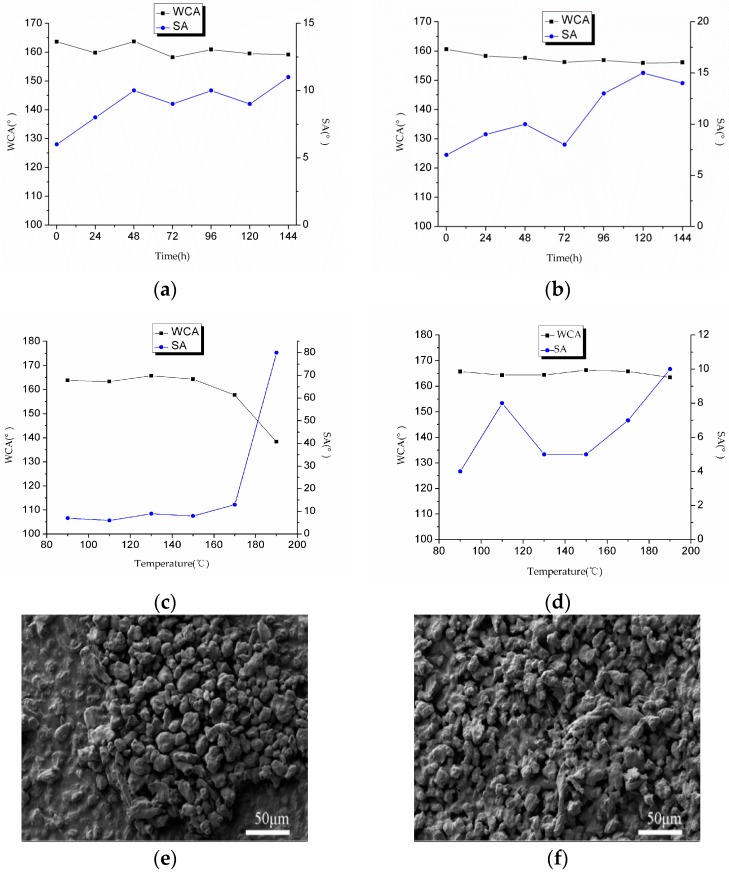
Change curves of the WCA and SA of (**a**) the D coating and (**b**) the E coating when exposed to UV radiation; change curves of the WCA and SA of (**c**) the D coating and (**d**) the E coating at different temperatures; surface morphologies of (**e**) the D coating and (**f**) the E coating after being fired with an alcohol burner for 1 min.

UV radiation is a very important detection index for durability. The D and E coatings were exposed to UV lamp radiation for six days (144 h), and the WCA and SA were measured every 24 h. As shown in Figure 7a,b, the results do not change significantly, which indicates that the L-CNC particles have a good UV radiation resistance and the chemical components on the particle surfaces show no change.

#### 2.5.2. Heat Resistance Test

Considering potential applications in some high-temperature locations, the superhydrophobic coatings need a heat resistance test. As shown in Figure 7c,d, the D and E coatings were tested at a variety of temperatures. The WCAs and SAs showed no obvious change even at 170 °C, proving that the coatings possess good heat stability, which benefits from the thermal stability of the adhesives and the L-CNC particles that are able to retain their morphologies. At 190 °C, the WCA of the D coating has an obvious reduction, but the E coating does not. To further test the thermal stability of the coatings, the bottom sides of the glass slides with the D and E coatings were fired above an alcohol burner for 1 min. The results show that the D coating loses its superhydrophobicity, but the E coating maintains this property. For the D coating, as shown in Figure 7e, the double-sided adhesive is melted, which causes some of the L-CNC particles to sink, resulting in damage of the surface structure. However, for the E coating, as shown in Figure 7f, the surface structure has no obvious change, which is attributed to the ultrahigh heat resistance of the epoxy.

#### 2.5.3. Acid and Alkali Resistance Test

**Figure 8:**
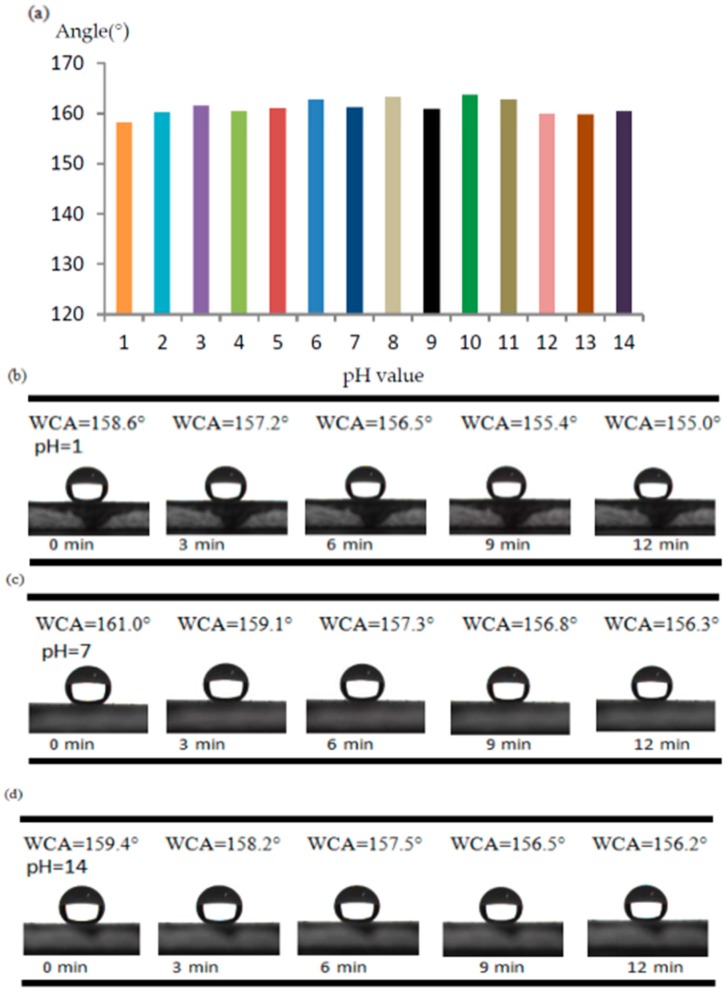
(**a**) WCAs of water droplets with different pH on the D coating; change in status of water droplets with (**b**) pH = 1; (**c**) pH = 7; and (**d**) pH = 14 over time on the D coating.

In the acid and alkali resistance tests, as shown in Figure 8a, the WCAs only showed a slight change, proving that the coatings could retain the surface structure and chemical components and that they had good resistance to acidic and alkali corrosion. To further test the coatings, water droplets with pH = 1, 7, and 14 were used to measure the WCA of the coatings; a measurement was taken every 3 min up to 12 min. The results are shown in Figure 8b,c; the WCA of the coatings decrease over time. At a water droplet with pH = 1, and the WCA shows only a reduction of 3.6° from 158.6° to 155.0° after 12 min. At a water droplet with pH = 14, it has only a reduction of 3.2° from 159.4° to 156.2° after 12 min. Lastly, at a water droplet with pH = 7, it also shows a similar reduction of 4.7° from 161.0° to 156.3° after 12 min. The reason for this small reduction of the WCAs is possibly that the gravity of the water droplets makes themselves sink after a long time, resulting in an increase of the contact area between the droplets and the coating surface. This demonstrates good acid and alkali resistance.

## 3. Materials and Methods 

### 3.1. Materials

BioPlus-L™ Crystals, a kind of lignin-coated cellulose nanocrystals (L-CNC) (brown powder, lignin content ~3–6 wt %, 4.3% moisture) were purchased from American Process Inc., Atlanta, GA, USA, and dried in an oven for two hours under 110 °C before use. The quick-setting epoxy (25 mL, two-component, clear, cures in an hour) was purchased from the J-B Weld company, Sulphur Springs, TX, USA. The double-sided tape (2’’ × 20 m, long-term hold, Hongxin Lattice) was purchased from Amazon, Seattle, WA, USA. Toluene (99.8%, extra dry, anhydrous) and 1H,1H,2H,2H-perfluorooctyltrichlorosilane (PFTS, CF_3_(CF_2_)_5_(CH_2_)_2_SiCl_3_, 97%) as a low energy reagent and anhydrous ethanol (200 proof) were purchased from Fisher Scientific, Waltham, MA USA. Glass slides (7.5 × 2.5 cm) were used as substrates and were washed for 5 min using distilled water in an ultrasonic cleaning machine and dried before use.

### 3.2. Preparation of L-CNC Surperhydrophobic Coating

#### 3.2.1. Hydrophobic Modification of L-CNC Particles

Two grams of L-CNC particles were added to 18 g of toluene and stirred for 10 min, followed by adding 1 g of PFTS and continuing to stir for 5 h. Then, the L-CNC particles were separated by a centrifuge and washed with anhydrous ethanol three times to remove the residual toluene and dissociative low-energy reagent, and finally were dried for 30 min at 60 °C.

#### 3.2.2. Quick-Setting Epoxy Modulation and Double-Sided Tape Handling

The two components of quick-setting epoxy were squeezed out with a quality ratio of 1:1 at the same time and then mixed and stirred for about 1 min. The double-sided tape was cut to the size of 2.5 cm × 2.5 cm.

#### 3.2.3. Hydrophobic L-CNC Particles and Adhesive Composite

The modulated epoxy was first smeared onto the substrate surface with a gluing surface of 2.5 cm × 2.5 cm, or the double-sided tape of 2.5 cm × 2.5 cm was stuck onto the substrate surface. Then, the adhesive surface was completely covered with the modified L-CNC particles scattered with a sieve (200 meshes), followed by rolling the particles with a smooth wooden bar for more than 1 min. The composite superhydrophobic coatings were successfully prepared.

### 3.3. Characterization

The chemical composition of the composite coating was analyzed using Fourier transform infrared spectroscopy (FTIR, Perkin-Elmer, Waltham, MA, USA, scanning 16 times in the range of 4000~600 cm^–1^). A Zeiss Auriga SEM/FIB crossbeam workstation (Oberkochen, Germany) was chosen to observe the surface morphology of the coating at an accelerating voltage of 3 kV.

**Figure 9:**
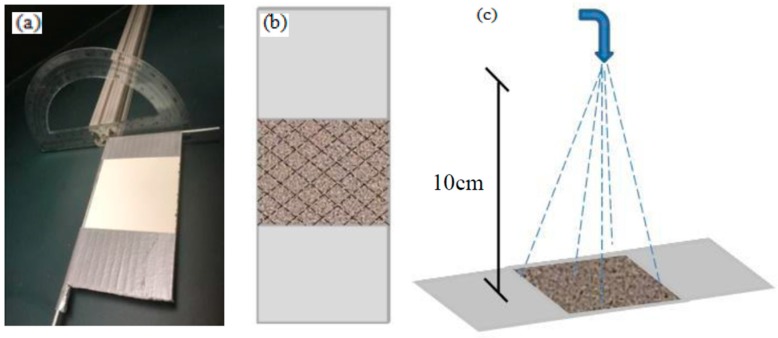
(**a**) Self-made slide angle measuring station; (**b**) knife-scratch and (**c**) water jet sketch.

WCA and SA measurement: The water contact angle (WCA) of the coating was obtained by a Drop Shape Analysis System (EasyDrop, Germany KRUSS, Hamburg, Germany). As reported in our previous paper [28], a self-made slide angle (SA) measuring station was built by simply inserting a “T” iron frame with a smooth panel into the superposed center between a protractor and a strip of hollow aluminum. As shown in Figure 9a, the smooth panel could be rotated by 180° and an SA value could be directly read from the protractor. Each WCA or SA was the average value of five measurements at different points using a 4~8 μL water droplet.

Self-cleaning: The coating with dust on its surface was tilted at a small angle, water droplets were added dropwise, and the situation of the coating surface was observed.

Water-repellent in oil: The coating was immersed into oil (n-dodecane). Then, water droplets were dripped dropwise onto the surface.

Sandpaper abrasion test: The method was similar to that in a previous report [34]. The coating against sandpaper (320 grit) was pressed with a 100-g weight and engaged a linear movement for 10 cm under external force. Then, the coating was horizontally wiggled at 90° and moved another 10 cm. This process was counted as one abrasion cycle.

Finger-wipe test: The sample was kept stationary by pressing with one hand, and was scraped with a finger from the other hand. This test was conducted three times in different areas.

Knife-scratch test: The sample was scratched with a knife along the intersecting lines as shown in Figure 9b, and then the WCA and SA were measured.

Water jet test: As shown in Figure 9c, the coating was tested using a water jet with a water flow rate of approximately 5 m/s, a water pressure of 50 kPa, and a standoff distance of 10 cm. The spray surface was approximately 40 cm^2^, and each jet last for 5 s. The WCAs and SAs were measured after each water jet, and the coatings were dried again.

Heat resistance test: The coating was placed in an oven with 90 °C, 110 °C, 130 °C, 150 °C, 170 °C, and 190 °C for 2 h, and then the WCAs and SAs were measured, respectively.

Acid and alkali resistance tests: The aqueous solution with pH 1-6 was deployed with 5 wt % HCl solution and the aqueous solution with pH 8-14 was deployed with 5 wt % NaOH solution. Then, the water droplets at pH 1 to 14 were dripped onto the coating surface and the WCAs and SAs were measured, respectively.

UV radiation test: The samples were continuously exposed under a Blak-Ray^®^ XX-15M UV bench lamp with a light source (UV-C LAMP G15T8/SW, power: 15 W, wavelength: 254 nm, UV micro watts: 49 uW/cm^2^ at 1 M, Sankyo Denki, Hiratsuka, Japan). The distance between the sample and the light source was 10 cm.

## 4. Conclusions

L-CNC particles are environmentally friendly and suitable to build rough surface structure for a superhydrophobic coating due to their irregular shape and surface morphology. A robust and durable superhydrophobic coating can be successfully prepared by a facile method in which the hydrophobic L-CNC particles are dispersed with a sieve on the adhesive (double-sided tape and quick-setting epoxy) surface and then rolled with a wooden bar. The resulted coatings show excellent superhydrophobic properties and water repellence in air or oil, also possessing outstanding mechanical strength against sandpaper abrasion, finger-wipe, knife-scratch, and water jet, as well as durability against high temperatures, acid and alkali solutions, and UV radiation. The D coating shows better sandpaper abrasion resistance and can be used in some places where abrasion resistance is the main consideration. Alternatively, the E coating possesses better water jet and heat resistance and is suitable for use in areas with high temperatures and heavy rain. In view of the environmental performance of the L-CNC particles and the simple preparation, the coatings are of great potential application value.
